# Out of phase: relevance of the medial septum for directional hearing and phonotaxis in the natural habitat of field crickets

**DOI:** 10.1007/s00359-013-0869-8

**Published:** 2013-11-27

**Authors:** Stefan Hirtenlehner, Heiner Römer, Arne K. D. Schmidt

**Affiliations:** Department of Zoology, Karl-Franzens-University, Universitätsplatz 2, 8010 Graz, Austria

**Keywords:** Directional hearing, Cricket, Acoustic tracheal system, Sound localization, Pressure difference receiver

## Abstract

A modified tracheal system is the anatomical basis for a pressure difference receiver in field crickets, where sound has access to the inner and outer side of the tympanum of the ear in the forelegs. A thin septum in the midline of a connecting trachea coupling both ears is regarded to be important in producing frequency-dependent interaural intensity differences (IIDs) for sound localization. However, the fundamental role of the septum in directional hearing has recently been challenged by the finding that the localization ability is ensured even with a perforated septum, at least under controlled laboratory conditions. Here, we investigated the influence of the medial septum on phonotaxis of female *Gryllus bimaculatus* under natural conditions. Surprisingly, even with a perforated septum, females reliably tracked a male calling song in the field. Although reduced by 5.2 dB, IIDs still averaged at 7.9 dB and provided a reliable proximate basis for the observed behavioural performance of operated females in the field. In contrast, in the closely related species *Gryllus campestris* the same septum perforation caused a dramatic decline in IIDs over all frequencies tested. We discuss this discrepancy with respect to a difference in the phenotype of their tracheal systems.

## Introduction

One of the most crucial tasks of sensory systems is the extraction of behaviourally relevant signals from the actual sensory scene (Ronacher et al. 2004) to reliably detect and localize signallers of interest, such as mates or predators (Brumm and Slabbekoorn 2005). The task of sound localization in small insects can be challenging due to imposed physical constraints in deriving sufficiently large interaural intensity differences (IIDs) between both ears. Field crickets with their rather pure tone calling songs and carrier frequencies (CFs) of around 5 kHz (Gerhardt and Huber 2002; Montealegre et al. 2011) are constrained by their relatively small size compared to the wavelength of sound, preventing the establishment of reasonably large IIDs through diffraction (Michelsen et al. 1994). At the same time, interaural time differences (ITDs) as directional cues are also very limited due to the small distance between the ears (Michelsen 1992; Robert 2005). The solution to this problem is the evolution of a pressure difference receiver, where sound has access to both sides of the ear via a modified tracheal system (for reviews see Lewis 1983; Michelsen et al. 1994; Michelsen 1992, 1998; Robert 2005). The tracheal system modified for sound transmission consists of two tracheal openings (spiracles) located at the lateral surface of the thorax, and a leg trachea that connects the spiracular opening to the inside of the tympanum on each side. In addition, a transverse trachea connects the tracheal arrangement of both sides. The inherent directionality results from three main sound components that act on the tympanum: an external ipsilateral component, an internal ipsilateral component through the ipsilateral spiracle, and a further internal component from the contralateral side (Hill and Boyan 1976, 1977; Larsen and Michelsen 1978; Fletcher and Thwaites 1979; Larsen 1981; Boyd and Lewis 1983; Michelsen et al. 1994). The latter component appears to be crucial for the establishment of sufficiently large IIDs. When sound is transmitted from the contralateral spiracle to the ipsilateral tympanum, it passes a thin central septum located in the middle of the transverse trachea. Sound transmission through the septum causes a phase delay only within a narrow range of frequencies (corresponding to the males calling song) and accordingly changes the phase relationship of ipsi- and contralateral sound components at the tympanum (Michelsen and Löhe 1995). In addition, the effect of a pressure difference receiver is enhanced by the fact that the propagation velocity of sound within the tracheal system is lower than in air, thus increasing the difference in arrival time of sound at the inner and outer side of the eardrum (Larsen 1981; Michelsen et al. 1994; Michelsen and Larsen 2008).

Laser vibratory measurements of the tympanum in *Gryllus bimaculatus* showed that the tuned directionality and the amount of IIDs were strongly decreased to 1–2 dB, from a peak of nearly 10 dB at a CF of 4.5 kHz, after destroying the medial septum (Michelsen and Löhe 1995). It has been argued that such a low amount of directional cues would not suffice for successful sound localization under field conditions (Michelsen 1998).

The behavioural relevance of the septum for female phonotaxis was tested in laboratory treadmill experiments by Wendler and Löhe (1993). They found that females of *G. bimaculatus* were still capable of locating a sound source after septum perforation. However, the error of angle towards the sound source increased to about 30°. In a subsequent study the role of the septum and its influence on directional properties of the auditory system of *G. bimaculatus* was studied in a neurophysiological approach. Recording summed receptor potentials of the leg nerve revealed IIDs of 14 dB at 5 kHz, which were reduced to about 7 dB after septum perforation (Löhe and Kleindienst 1994).

Although the effect of septum perforation on female phonotaxis has been investigated in a controlled laboratory setting within a homogenous sound field (Wendler and Löhe 1993), the role of the medial septum for sound localization under field conditions, as suggested by Michelsen (1998), is still unclear. However, differences in the outcome between laboratory experiments and those conducted outdoors should be expected (Bee and Micheyl 2008; Schmidt and Römer 2011). In a field study on acoustic preferences of female *G. bimaculatus*, Hirtenlehner and Römer (unpublished) found that differences in carrier frequency, intensity and chirp rate of the calling song needed to be larger for a significant preference compared to results under laboratory conditions. Several factors such as degradation of directional cues, species-specific amplitude modulations (AM) and non-linear intensity gradients over distance may have contributed to this difference in the observed behavioural performance (Römer 1998, 2001; Kostarakos and Römer 2010; Bradbury and Vehrencamp 2011). Furthermore, females are not completely free to walk towards a sound source in their natural environment, but have to circumvent obstacles in the transmission channel. These forced changes in walking direction could bias a phonotactic track more than the turns induced by the sensory information. A neurophysiological study outdoors on *G. bimaculatus* revealed a highly fluctuating directional and amplitude gradient on the transmission channel with positions where the directional information or AM modulation of the calling song was not available (Kostarakos and Römer 2010). Thus, it is not known how field crickets deal with such a complicated situation when they experience a loss of directional cues after septum perforation. Under such conditions, one would expect that phonotactic performance and the overall ability of sound source localization is severely impaired.

Here we studied the importance of the medial septum for sound localization and phonotactic behaviour of female *G. bimaculatus* in their natural habitat. Phonotactic trials were conducted with the same individuals before and after septum perforation. The result of the manipulation was quantified both anatomically and physiologically by measuring the tuned directionality of the peripheral auditory system using neurophysiological methods. The effect of septum manipulation for directional tuning of the ear was compared with a closely related cricket species (*G. campestris*) and results are discussed with respect to the structural differences of the tracheal anatomy.

## Materials and methods

### Animals and study site

Adult field crickets (*Gryllus bimaculatus* de Geer) were reared at the Department of Zoology, University Graz. The cricket population was kept at an ambient temperature between 25 and 30 °C at a constant photo cycle of 12:12 h L:D and fed ad libitum on a diet of water, fish flakes, oats and lettuce. To maintain phonotactic responsiveness, females were separated from males starting as last instars; behavioural experiments were conducted 1 week after their final moult.

Outdoor experiments were carried out in a natural grassland area in the vicinity of Graz, Austria (47°6′1.2348″N; 15°27′9.7272″E) in August/September 2012 and June/July 2013. The experimental area consisted of a patch of meadow (5–10 cm in height) covering ~3.4 qm^2^. We performed phonotaxis experiments constantly at the same time of day between 2 and 5 p.m. Temperature at the walking position of crickets ranged between about 20 and 25 °C. Behavioural experiments were conducted exclusively with female *G. bimaculatus*. For a comparative analysis of the change of IIDs, we performed neurophysiological experiments on females of *G. bimaculatus* as well as on *G. campestris* L. All individuals of *G. campestris* were wild caught in Styria, Austria.

### Acoustic stimulation

Acoustic stimuli were digitally generated at a sampling rate of 48 k samples s^−1^ using software Cool Edit Pro (version 2.0; Syntrillium, Phoenix, AZ, USA). The temporal pattern of a conspecific chirp consisted of four consecutive pulses of 23 ms duration separated by a constant interpulse interval of 16 ms, resulting in a chirp duration of 140 ms. Chirps were repeated every 400 ms (i.e. 150 chirps/min) at a CF of 4.9 kHz, the average best frequency (BF, i.e. frequency of lowest threshold) of female receivers (Kostarakos et al. 2008; Hirtenlehner et al. 2013). Stimuli were broadcast using an MP3 player (Bogieman IV, X4-Tech, Braunschweig, Germany) connected to an amplifier (Kemo-Electronic, M033, Langen, Germany) and a speaker (Monacor Electronic, SP-626/8, Zwischenwasser, Austria; frequency response 600–18,000 Hz). Sound stimuli were calibrated to 85 dB SPL (sound pressure level) at a distance of 0.5 m (Rion NL-21 sound level meter and UC-52 ½ inch free-field microphone, Tokyo, Japan).

### Experimental procedure

To track the female phonotactic path, we mounted a video camera (SONY handycam HDR-XR155E, Minato, Tokyo, Japan) onto a custom-made sliding holder attached to a steel rope at a height of 2.5 m above the experimental area. Video recordings were used for off-line analysis of phonotactic approaches. During phonotaxis, the experimenter was positioned 5 m away from the test area and manually controlled the match of the camera’s display detail and the cricket’s position via a monitor (SONY Video Walkman GV-D900E PAL, Minato, Tokyo, Japan) connected to the video camera sliding device above the walking female.

The two speakers were positioned on the ground and separated by 160 cm from each other at a distance of 200 cm from the release point of the female such that the two sound sources and the release point confined an angle of nearly 55°.

We tested each *G. bimaculatus* female with an intact medial septum in no-choice trials for control and re-tested the same individuals under identical stimulus conditions after perforating their medial septum (for operational procedure of septum perforation, see below). Females were kept in plastic boxes at the release point for half-an-hour to adapt to the experimental area. Ten minutes before testing, females were exposed to low SPLs of calling songs. Females were tested in no-choice trials by broadcasting the sound stimuli from either of the two speakers. To avoid a potential side bias in the outdoor setup or asymmetries in the sound propagation due to physical conditions of the grassland transmission channel, we retested the females by using the alternative speaker. Importantly, we also randomized the position of the starting point on the test field within a range of 6 m day by day. Thus, females did not experience exactly the same transmission channel, such as patches of grass bundles, in a new trial.

Test trails were initiated by releasing the female from the box and started as soon as the female began walking. A phonotactic approach was counted successful when the female reached a semicircle with a radius of 10 cm around the centre of a speaker, although 90 % of females directly mounted the speaker. Females that did not show phonotaxis on three consecutive days were excluded from further experiments.

### Data analysis

Video files of phonotactic tracks were converted from MTS type to AVI using Any Video Converter (version 3.3.3), and two frames per second of the recordings were taken for later analysis using VirtualDub (version 1.9.11). Frames were imported in ImageJ 1.44p (Rasband 1997–2011), which allowed a precise frame by frame identification of x- and y-positions of a female via MTrackJ plugin (Meijering et al. 2012). Thus, the complete phonotactic approaches could be reproduced in Microsoft Excel. Statistical analysis was performed in SigmaPlot (version 12.03; Systat Software Inc., Chicago, IL, USA). To analyse differences in the various parameters of phonotactic paths before and after the septum perforation, we used the mean values of two observations for each individual. To test if perforation of the medial septum significantly affects the walked detour, the vector length, the time needed for covering the walked distance and the walking speed of the same individuals, we performed paired *t* tests, after evaluating the data by performing Shapiro–Wilk normality test.

### Neurophysiological experiments

To quantify the change of IIDs before and after the septum manipulation, we examined the directional tuning of the ear physiologically, using the ascending neuron 1 (AN1), as an indicator (Kostarakos et al. 2008). Details of the preparation technique are described elsewhere (Stabel et al. 1989; Kostarakos et al. 2008). We defined the neuronal threshold as the minimum stimulus intensity which elicited at least one action potential per syllable. Acoustic stimuli (see above) were varied in their CF ranging from 3 to 6 kHz, in steps of 100 or 500 Hz (see Fig. 3). Song models were amplified (custom-made high-frequency amplifier), attenuated in steps of 1 dB using a digital attenuator (PA5, Tucker Davis Technologies, Alachua, FL, USA) and broadcast via a speaker (Raveland, MHX 138) positioned at a distance of 40 cm in front of the preparation.

To quantify IIDs, we calculated the threshold differences between ipsilateral and contralateral stimulation at an angle of 30° off the longitudinal body axis in the horizontal plane to the preparation. Neurophysiological experiments were conducted at room temperature (about 22 °C).

A sound pressure level of 85 dB used in outdoor experiments is the amplitude range of natural cricket songs measured in the field (own measurements); this level is about 40 dB above the neuronal threshold of *G. bimaculatus*. The behavioural threshold, defined as the SPL at which females start orienting towards a sound source is about 40–50 dB (Popov and Shuvalov 1977), but phonotaxis performance is considerably improved at values between 70 and 80 dB SPL (Wendler and Löhe 1993). Furthermore, 85 dB SPL (as calibrated at a distance of 0.5 m corresponds to 62 dB SPL at the release point of female crickets on the test ground. Therefore, in their approach to the active speaker, females move within the dynamic range of intensity function of AN1, which provides the afferent information about the calling song in the brain (Schildberger and Hörner 1988).

### Septum perforation

After intact completion of phonotactic experiments, non-anesthetized females were carefully fixed ventral side up with soft modelling clay under a binocular microscope (Wild M10, Leica, Wetzlar, Germany) in such a way that all legs were immobilized and a human hair could be inserted through the spiracle beyond the forelegs, thus perforating the medial septum (Wendler and Löhe 1993). After retesting phonotaxis in the field (carried out at least 1 day after the operational procedure) and evaluation of directional tuning in neurophysiological experiments, the acoustic vesicle (i.e. the enlarged part in the midline of the transverse trachea accompanied with the septum; see above) with the medial septum was dissected for visual control. Those animals whose tracheal branch (i.e. transverse trachea) was accidentally perforated were excluded from the data set. The same procedure of septum perforation was carried out in neurophysiological experiments. Overhead shots of the septum were taken using a digital microscope camera (DCMC 510, 5 M pixels, Oplenic Optronics CO., LTD, Hangzhou, China) and the magnitude of destroyed septum area was calculated as percent of total septum area using ImageJ. The same procedure was carried out in neurophysiological experiments for evaluation of IIDs.

## Results

### Behavioural experiments

**Fig. 1:**
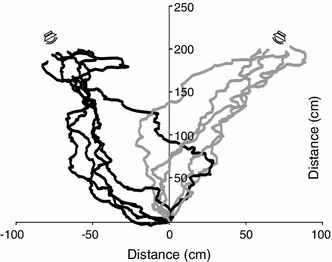
Phonotactic tracks of the same five individual females (*Gryllus bimaculatus*) in the natural habitat before (*grey lines*) and after (*black lines*) septum perforation. Sound localization was not significantly impaired after the septum was destroyed. For illustration purpose, tracks for females with the intact septum are shown on the *right side* and with perforated septum on the *left side* (for more details see text)

**Fig. 2:**
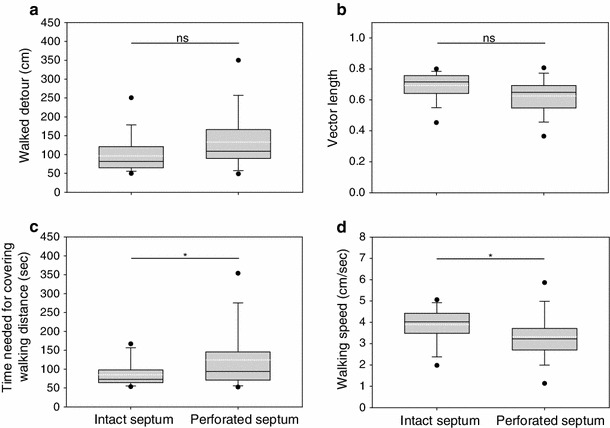
Behavioural performance of female *G. bimaculatus* in no-choice experiments under natural conditions (*box plots black line* = median; *white dashed line* = mean; whiskers above and below the box indicate 90th and 10th percentiles, respectively)

Fifteen intact females of *G. bimaculatus* that successfully approached the sound source also reliably tracked the speaker after the destruction of their medial septum. The comparison of walking paths of intact and septum-perforated females (Fig. 1; for clarity, the paths of only five representative females are shown) indicates that the latter approached the active speaker in a similar way as intact females. A quantitative analysis showed that intact females walked an average detour of 96.1 ± 12.9 cm (mean ± SEM) ranging from 49.8 to 250.6 cm, whereby detour was calculated as the difference between the actually walked path length minus the linear distance from an animals’ starting position to the active speaker. Destruction of the medial septum resulted in a slight, but not significant increase of covered detour: when approaching the active speaker, operated females walked detours as large as 132.65 ± 19.3 cm to cover the shortest path of the 200 cm test trial (paired *t* test: *t* = −1.478, *N* = 15, *P* = 0.162; Fig. 2a). The straightness of walking paths can also be evaluated by computing the vector length (VL) as a ratio of the length of the shortest distance divided by the length of the actual walked path (Wendler and Löhe 1993). The mean VL value of 0.695 for phonotactic tracks performed by intact females did not differ significantly from 0.627 for walking paths of operated females (paired *t* test: *t* = 1.693, *N* = 15, *P* = 0.113; Fig. 2b).

On average, intact females arrived at the active speaker 84.85 ± 8.6 s after being released at the starting point, whereas after the perforation of their medial septum females needed significantly more time (123.22 ± 20.2 s) to cover the distance to the speaker (paired *t* test: *t* = −2.339, *N* = 15, *P* = 0.035; Fig. 2c). The females’ walking speed was affected in a similar way (see Fig. 2d): Females with an intact medial septum covered a distance of 3.89 ± 0.21 cm/s in the natural habitat, but after the operation the same females walked with a reduced average speed of 3.32 ± 1.05 cm/s (paired *t* test: *t* = 2.866, *N* = 15, *P* = 0.012).

### Neurophysiology

To evaluate the effect of septum perforation on the tuned directionality of the cricket ear, IIDs and the sensitivity tuning of AN1 were determined for the two species, *G. bimaculatus* and *G. campestris*.

### *Gryllus bimaculatus*

**Fig. 3:**
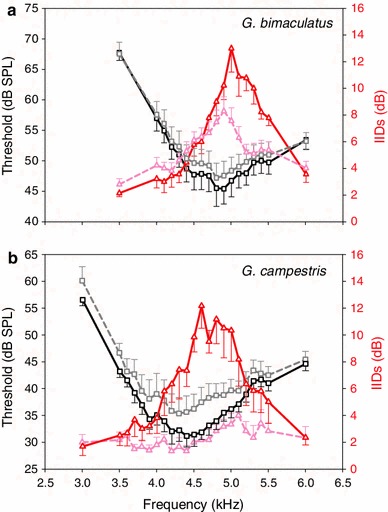
Comparison of sensitivity and directional tuning in *G. bimaculatus* **a** and *G. campestris* **b** before and after septum perforation. *Squares* indicate sensitivity tuning, whereas triangles indicate directional tuning of intact (*solid line*) and operated (*dashed line*) females

**Fig. 4:**
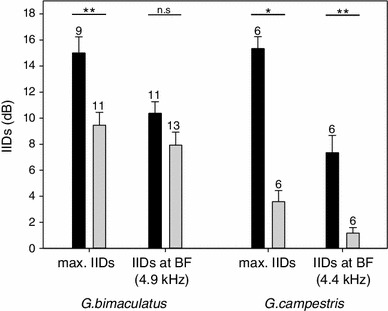
Effect of septum perforation on the magnitude of maximum IIDs and best frequency of 4.9 kHz for *G. bimaculatus* and 4.4 kHz for *G. campestris* (*black bars* = intact septum; *grey bars* = perforated septum; numbers indicate sample size)

The sensitivity tuning of AN1 in animals with an intact septum revealed a BF at 4.9 kHz and a threshold of 45.4 dB SPL (Fig. 3a, black squares and solid line), whereas septum perforation leads to a 100 Hz frequency shift with a BF at 4.8 kHz and a threshold of 47.1 dB SPL (Fig. 3a, grey squares and dashed line). Septum manipulation did not change the AN1 tuning significantly; however, a slight loss of sensitivity of 2 dB for frequencies between 4.5 and 5.2 kHz was observed. We found a notable effect of septum perforation on the directional tuning of the auditory system and the amount of IIDs (Fig. 4). Thus, average maximum IIDs decreased from 15 ± 1.24 dB (individual values ranging between 10 and 22 dB) to 9.45 ± 0.99 dB (individual values ranging between 3 and 15 dB) and were significantly reduced on average by 5.22 ± 1.85 dB (signed rank test: *Z* = −2.52, *P* = 0.008, *N* = 9/11). However, the average IIDs at 4.9 kHz, the song frequency at which behavioural outdoor experiments were performed, were not significantly different between the intact and perforated septum (paired *t* test: *t* = 1.425, *P* = 0.185; *N* = 11/13) with values of 10.36 ± 0.90 dB (range of IIDs from 5 to 16 dB) and 7.92 ± 1.0 dB (range of IIDs from 2 to 15 dB), respectively.

**Fig. 5:**
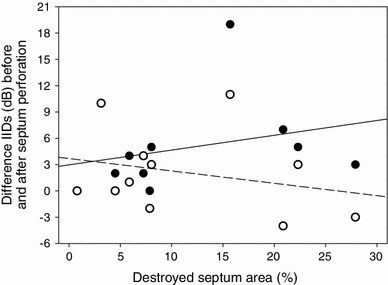
Degree of septum destruction and the change of IIDs in *G. bimaculatus*. For different individuals the maximum IIDs (*open circles*, *N* = 11) and IIDs at 4.9 kHz (*solid circles*, *N* = 9) were considered. In both situations, regression coefficients were low with *R* ^2^ = 0.07 and no significant linear relationship was found

We observed a shift in the BF of directional tuning (5 kHz) by 110 Hz towards lower frequencies after destroying the medial septum (4.89 kHz); in single preparations the shift could increase to 500 Hz. There was no significant linear relationship in the correlation between the size of the destroyed septum area and the difference of IIDs measured in individuals with an intact and a destroyed septum, when considering maximum IIDs (Fig. 5, *R* ^2^ = 0.07, Spearman coefficient = 0.496, *P* = 0.177) or IIDs at 4.9 kHz (*R* ^2^ = 0.07, Spearman coefficient = −0.224, *P* = 0.508; Fig. 5). The dimension of septum perforations ranged between 0.8 and 27.9 % of the total septum area, with an average of 10.6 ± 2.3 %.

### *Gryllus campestris*

Sensitivity tuning of AN1 and the directional tuning were also measured in six individuals of *G. campestris.* With an intact system the BF of average frequency sensitivity was 4.4 kHz with a threshold of 31.1 dB SPL, which changed to 4.3 kHz with a threshold of 35.3 dB SPL when the septum was perforated. The operation resulted in a somewhat higher loss of sensitivity compared to *G. bimaculatus*, but more importantly, the directionality of the ear changed dramatically (Fig. 3b). Maximum IIDs were significantly reduced from 15.3 ± 0.9 dB to only 3.6 ± 0.9 dB (Signed rank test: *Z* = −2.23, *P* = 0.03, *N* = 6). IIDs at the species’ BF of 4.4 kHz (Kostarakos 2009) were significantly reduced from 7.3 ± 1.2 to 1.3 ± 0.4 dB (paired *t* test: *t* = 4,954, *P* = 0.004; *N* = 6; Fig. 4). Over the whole range of tested frequencies, there was no indication of a tuned directionality left after septum perforation. The dimension of septum perforations ranged between 0.8 and 16.4 % of the total septum area with an average of 10.8 ± 2.7 %.

## Discussion

The evolution of a sophisticated pressure difference receiver in crickets, with a complex anatomical arrangement of connecting trachea, appears to be essential for the frequency-tuned directionality of the ears and the ability of receivers to localize a sound source (Michelsen 1998; Gerhardt and Huber 2002; Robert 2005). Here, we evaluated the role of the medial septum for directional hearing under natural conditions outdoors, after Wendler and Löhe (1993) had shown that perforation of the septum caused changes in essential characteristics of female phonotaxis, and average IIDs were markedly reduced (Löhe and Kleindienst 1994).

### Behavioural outdoor experiments

Contrary to our assumption, septum perforation had only little influence on the localization ability of female *G. bimaculatus.* Phonotactic tracks did not change significantly compared to those of intact females with respect to walked detours and vector lengths (Fig. 2). The only phonotactic parameters affected by the operation was the significantly increased time needed to cover the distance to the target and the decreased speed of walking. One could interpret this finding by the need to compensate the reduced directional information of 2 dB at the frequency of 4.9 kHz (where all phonotaxis experiments have been performed) after septum perforation with integration over longer periods of time for making correct directional decisions.

The results obtained in laboratory treadmill experiments showed a clear difference between intact and perforated females, where for the latter all aspects of phonotaxis performance (steering accuracy, walking speed, etc.) were deteriorated (Wendler and Löhe 1993). Since we analysed vector length and walking speed as well, we can directly compare the data obtained under natural conditions and in the laboratory. Vector length of intact females was considerably higher in laboratory treadmill experiments compared to outdoors with values of 0.85 and 0.69, respectively. The fact that females walked less straight towards the sound source under field conditions is an obvious consequence of the highly structured grassland, where females cannot walk straight towards the sound source, but are forced to make turns due to various obstacles. On the other hand, with the septum perforated the difference in vector length was less pronounced with values of 0.63 and 0.65 under natural and laboratory conditions, respectively. Thus, whereas on the treadmill the perforation reduces the effectiveness of the phonotactic approach, it does not outdoors, because the structural nature of the transmission channel does not allow a better performance. The same argument could also hold for the strongly reduced walking speed for both intact and operated females in the grassland habitat compared to experiments conducted on the walking compensator, if we consider that females are forced to bypass obstacles on a highly disturbed track.

Hirtenlehner and Römer (unpublished) observed that in their natural habitat female crickets are not capable of distinguishing small differences between song traits, which they can detect in undisturbed soundproof rooms. Physical conditions of sound transmission in the natural grassland differ substantially compared to the homogenous sound field of trackball and treadmill experiments in the laboratory (Michelsen 1978; Embleton 1996; Römer 1998). The local directional information at the position of a receiver can be strongly impaired (Rheinlaender and Römer 1986; Gilbert and Elsner 2000; Kostarakos and Römer 2010). In addition, signal attenuation and degradation on the transmission channel will further reduce sensory information for a receiver (Wiley and Richards 1982; Römer and Lewald 1992; Römer 1998; Bradbury and Vehrencamp 2011). Therefore, it can be expected that overall differences in phonotactic parameters between intact and manipulated females are less important under field conditions.

### Sufficient directional information after septum perforation in *G. bimaculatus*

The fact that the phonotactic performance of septum-perforated female *G. bimaculatus* was almost unimpaired in the field suggests that sufficient binaural directional cues must have been available for sound source localization. Indeed, the analysis revealed that the directionality of the ear after septum perforation was in good agreement with the behavioural data. At 4.9 kHz, the song frequency at which the average receiver is tuned, the average IIDs were as high as 7.9 ± 1.0 dB and thus similar to values of 7 dB obtained by Löhe and Kleindienst (1994). If phonotaxis is based on the decision rule “turn to the side more strongly stimulated” (Fraenkel and Gunn 1961; Weber and Thorson 1988, 1989; but see Wendler 1989; Stabel et al. 1989 for another decision rule “*turn towards the better pattern*”), then the amount of 8 dB in IID should indeed be sufficient even under outdoor conditions. The amount of IIDs is highly correlated with the instantaneous bilateral discharge differences of the pair of AN1 neuron relevant for phonotaxis, and IIDs of only 6 dB will translate into response differences of about 6 APs/chirp. Such differences result in a strong phonotactic response of female crickets towards a sound source in no-choice trials on a trackball system (Kostarakos 2009), and even for two-choice experiments it is far above the threshold difference of 2.0 APs/chirp necessary for a significant steering towards one of the alternative song model (Trobe et al. 2011; Hirtenlehner et al. 2013). Thus, our observation of a successful phonotactic performance of septum-perforated females is in accordance with a remaining IID of 7.9 dB at 4.9 kHz obtained in neurophysiological experiments.

One may argue that the amount of septum perforation was not enough to achieve a reduction in IIDs, which significantly reduced behavioural performance. However, the dimension of induced septum perforations varied between 1 and 28 % of the total area, and were thus within the range obtained in previous studies (Wendler and Löhe 1993: 2–11 %; Löhe and Kleindienst 1994: 2–10 %; Michelsen and Löhe 1995: 10–25 %). More importantly, we found no correlation between the destroyed septum area and a change in the magnitude of IIDs reduction in females of *G. bimaculatus* (Fig. 5). Obviously, even a minor damage of the septum appears to alter its vibration characteristics and/or a puncture in the septum may allow an exchange of air of the two tracheal compartments, so that the propagation media for the sound waves are not principally separated anymore. A mixture of air due to the loss of this physical separation may probably contribute to a loss of a phase shift effect. As a result, maximum IIDs were reduced on average by 5.5 dB from 15 to 9.5 dB (Fig. 4), similar to values obtained by Löhe and Kleindienst (1994) of 7 dB. Despite the septum manipulation, the directionality of the ear was still tuned to a particular frequency, although with a shift in BF of directional tuning by 100 Hz towards lower frequencies (Fig. 3a).

The fact that sufficiently large IIDs can be achieved in *G. bimaculatus* even without the medial septum functioning as a mechanical phase shifter might challenge its integrity for directional hearing. The importance of the septum has been demonstrated by Michelsen and Löhe (1995) where the tuned directionality was completely eliminated (IIDs between 0 and 2.5 dB in a frequency range of 3.5–5.5 kHz). However, such a dramatically reduced amount of IIDs obtained for the single individual documented might be the exception rather than the rule, at least in *G. bimaculatus*. Indeed, we observed such a strong reduction in only 1 out of 11 preparations, whereas IIDs amount on average 7.5 dB at 4.9 kHz and thus match well with the value of 7 dB obtained by Löhe and Kleindienst (1994) at about the same frequency.

### Differences in directional tuning of *G. bimaculatus* and *G. campestris*

Thus, how important is the medial septum for directional hearing? Previous behavioural data already indicated that acoustic coupling of both ears seemed not absolutely necessary for sound source localization (Weber and Thorson 1989) implying that the pressure difference receiver could principally function like the one found in katydids where sound transmission to the inner and outer tympanum is restricted to each body side (Robert 2005).

**Fig. 6:**
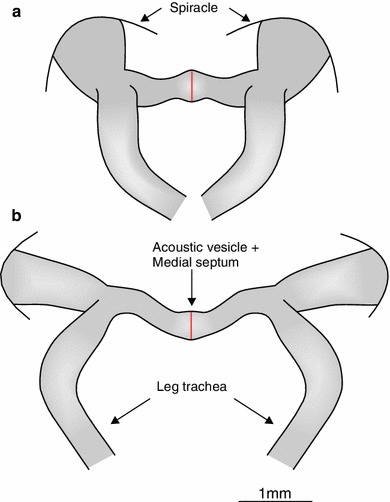
Comparison of the acoustic tracheal system in *G. bimaculatus* **a** and *G. campestris* **b**. Morphological differences between both systems such as the somewhat enlarged spiracular opening may exhibit effects on sound transmission within the tracheal system that lead to the observed differences in the amount of IIDs

Surprisingly, the same kind and amount of destruction of the septum in the field cricket *G. campestris* resulted in almost complete elimination of directionality. Septum perforation reduced the average IIDs by more than 11 dB and thus twice as much as observed for *G. bimaculatus*. The effect of the medial septum on sound transmission and the generation of IIDs appears to be much stronger in *G. campestris* compared to *G. bimaculatus*. What can account for this species-specific dependency in the way IIDs are produced? Because both species are of similar size, no diffraction pattern higher than 1–2 dB observed for *G. bimaculatus* can be expected (Michelsen et al. 1994; Michelsen 1998). Furthermore, the magnitude of septum destruction between both species was very similar with values on average of 10.3 % (*G. bimaculatus*) and 10.6 % (*G. campestris*). The anatomy of the acoustic tracheal system between both species reveals structural differences that may account for the observed difference and contribute to sound transmission (Fig. 6). Particularly, the tracheal diameter close to the external sound entrance of the spiracle is considerably expanded in *G. bimaculatus*, somewhat analogous to the exponential horn in katydids to enhance the gain of the internal sound pressure component to the ear (Lewis 1983). However, it is still unclear to what extent such a differently shaped acoustic trachea could enhance directional hearing in concert with the action of the septum. Unfortunately, since individuals of *G. campestris* used in our study were wild caught later in the season, no phonotactically responsive females were on hand to test the behavioural performance with a system of highly degraded binaural directional cues induced after the septum perforation. Thus, it is still an open question what the minimum amount of IIDs would be to enable reliable sound source localization under natural conditions.

### The acoustic tracheal system in an evolutionary context

In a recent study evaluating the diversity of acoustic tracheal systems in 40 cricket species and species of other related taxa of Ensifera, we found a strong association in the presence of an acoustic vesicle and a medial septum with the emergence of intraspecific acoustic communication (Schmidt and Römer 2013). Species that secondarily had lost acoustic signalling or those that primarily never used acoustic advertisement calls lack those key structures. Moreover, cricket species with an unfavourable ratio of body size to sound wavelength, critical for the generation of binaural directional cues via diffraction (Morse and Ingard 1969; Robert 2005), tended to exhibit a larger acoustic vesicle and medial septum. Those findings emphasize the importance of the acoustic vesicle and the medial septum for the task of sound localization and hearing and its potential role in the evolution of directional hearing.

## Conclusion

For the field cricket *G. bimaculatus*, we showed a clear effect of the medial septum for the magnitude of IIDs (5.5 dB on average); however, a reduction of IIDs by this amount had no consequences for the phonotactic approach to a single sound source under natural conditions. The behavioural performance can be explained by the relatively high IIDs initially provided in the intact system assuring sufficient binaural directional cues even after septum destruction and associated decrease of IIDs. In contrast, for *G. campestris* septum destruction had severe consequences regarding the amount of interaural intensity differences and tuned directionality.
